# The Role of Hepcidin in Myelodysplastic Syndromes (MDS): A Systematic Review of Observational Studies

**DOI:** 10.3390/cancers16020332

**Published:** 2024-01-11

**Authors:** Artur Słomka, Anna Pokrzywa, Dominika Strzała, Maja Kubiaczyk, Oliwia Wesolowska, Kinga Denkiewicz, Jan Styczyński

**Affiliations:** 1 Department of Pathophysiology, Nicolaus Copernicus University in Toruń, Ludwik Rydygier Collegium Medicum in Bydgoszcz, 85-094 Bydgoszcz, Poland; 288452@stud.umk.pl (A.P.); 306211@stud.umk.pl (D.S.); 287261@stud.umk.pl (M.K.); 301637@stud.umk.pl (O.W.); 301685@stud.umk.pl (K.D.); 2 Department of Pediatric Hematology and Oncology, Nicolaus Copernicus University in Toruń, Ludwik Rydygier Collegium Medicum in Bydgoszcz, 85-094 Bydgoszcz, Poland; jstyczynski@cm.umk.pl

**Keywords:** iron, hepcidin, myelodysplastic syndromes

## Abstract

**Simple Summary:**

In our systematic review, we analyzed and summarized observational studies revealing a potential association between myelodysplastic syndromes (MDS) and hepcidin. The extensive studies available in this area enabled us to draw the conclusion that hepcidin has a potential importance in the pathophysiology of MDS and the prediction of poor MDS patient outcomes. A summary of the mechanisms leading to iron overload in MDS and the potential causes of elevated serum hepcidin levels are shown in a graphical abstract created with BioRender. It is important to note that this systematic review is based on a relatively small number of MDS patients and control participants. Hence, additional research is crucial for the further exploration of these findings.

**Abstract:**

Iron overload emerges as a serious complication in myelodysplastic syndromes (MDS), particularly associated with frequent transfusions during the course of the disease. The discovery and description of hepcidin’s mechanisms of action have contributed to a deeper understanding of iron metabolism. The existing literature reports a potential role of hepcidin in MDS, yet these data are fragmented and presented in an unstructured, somewhat chaotic manner. Hence, to address the existing data, we performed a systematic review of observational studies examining hepcidin levels in MDS. An extensive review of three bibliographic databases (Pubmed, Web of Science, and Scopus) enabled us to identify 12 observational studies. These studies focused primarily on adult patients with low-risk MDS who underwent transfusions and chelation therapy. An in-depth analysis of these manuscripts led to four main conclusions: (1) although high serum hepcidin levels are associated with MDS, most studies generally have not found a significant difference in these levels between patients and healthy individuals; (2) serum hepcidin levels are specific to MDS type; (3) serum hepcidin levels in MDS are strongly associated with transfusions and the genetic status of patients; and (4) high-risk MDS is associated with high serum hepcidin levels. While we have furnished a comprehensive summary of the significance of hepcidin in MDS, there are still gaps that future research should address. This pertains primarily to the capacity of hepcidin in predicting adverse outcomes for MDS patients and evaluating the efficacy of chelation therapy or the need for transfusion.

## 1. Introduction

Myelodysplastic syndromes (MDS) are a group of clonal hematologic neoplasms characterized by impaired hematopoietic stem cell (HSC) differentiation and inefficient hematopoiesis in the bone marrow [1,2]. In the United States, there are approximately 4 per 100,000 new cases reported annually and in Europe, this number is believed to be twice as high [3,4]. The heterogeneity of the disease and its remarkably intricate pathophysiology mean that, despite advancements in understanding the molecular basis of MDS, its etiology is known in only about 15% of cases [5]. Numerous studies have evaluated links between the disease and both genetic and environmental factors. However, it is widely acknowledged that advanced age and previous anticancer therapy significantly increase the risk of MDS [6,7].

A clinically substantial phenomenon observed in the course of MDS which affects the attainment of therapy and patient survival is iron overload (IO) [8,9,10,11]. The lack of iron utilization due to ineffective erythropoiesis and frequent blood transfusions in MDS patients results in IO, which portends a worse prognosis, including leukemia-free survival and overall survival [12,13]. It is therefore unsurprising that iron chelation therapy has a positive impact on the survival of patients with MDS [14,15], as confirmed in a meta-analysis conducted on a sizable cohort of 1562 patients diagnosed with this disease [16]. However, the intricacies of IO in the course of MDS may be much more nuanced than they appear at first glance. In fact, a complex molecular machinery orchestrates various biological processes controlling iron metabolism in MDS. 

As previously mentioned, the regulation of iron metabolism relies on a sophisticated mechanism [17]. However, for the past two decades, the prevailing postulation has suggested that hepcidin is the cardinal hormone responsible for maintaining iron balance in the body [18]. Produced by hepatocytes, the protein deactivates the sole known cellular iron exporter, ferroportin, thus inhibiting iron absorption and iron release from its cellular compartments [19]. The primary factors behind hepcidin synthesis stimulation are high iron stores, inflammation, and infection; thus, it is natural to delve into the role of hepcidin in the pathophysiology of IO, especially in the course of MDS. Recent intensive research has revealed the involvement of numerous proteins in regulating hepcidin synthesis and systemic iron metabolism [18,19]. One particularly intriguing player is erythroferrone (ERFE), known for its role in inhibiting hepatic hepcidin synthesis [18,19]. It is essential to explore the link between ineffective bone marrow erythropoiesis in MDS and the impact of ERFE and hepcidin on causing iron overload. ERFE, derived from erythropoietin (ERO)-stimulated erythroblasts [19], plays a pivotal role in understanding this connection.

A variety of works concentrating on hepcidin levels and MDS have been published [20,21,22,23,24,25,26,27,28,29,30,31]. Nonetheless, these studies do not present a unified concept regarding the role of hepcidin in MDS; hence, there is a need to systematize our knowledge in this field. Some studies describe a potential relationship of hepcidin with IO in the course of MDS [20,22], or the impact of MDS treatment on hepcidin levels [24,27], but these data are only provided fragmentarily.

In a previous publication, we explored the role of hepcidin in acute leukemias [32]. However, as far as we are aware, there is still no systematic review that completely addresses the role of hepcidin in MDS. In order to shed light on said knowledge gap, we conducted an extensive review of various observational papers detailing hepcidin levels in patients with MDS. In this manuscript, our primary point of focus is to assess the association of hepcidin with MDS, including the type of therapy used as well as the subtype of MDS or its severity. Our aim is to provide a cohesive systematic review of hepcidin’s role in MDS, consolidating and summarizing the findings revealed by other authors.

## 2. Materials and Methods

### 2.1. Search Methodology

We conducted a systematic review following the Preferred Reporting Items for Systematic Reviews and Meta-Analyses (PRISMA) statement [33] (Supplementary Table S1). Additionally, we registered this review in the International Prospective Register of Systematic Reviews (PROSPERO identifier: CRD42023404447) [34].

We performed a systematic search using online databases such as PubMed, Scopus, and Web of Science Core Collection to identify studies published up until the fourth of November 2022 (date of the last search). The number of these databases ensured sufficient coverage, reducing the probability of overlooking any relevant research [35]. The full PubMed search strategy is provided in Supplementary Table S2 and has been adequately translated for the other two databases. Our specific search terms were: “hepcidin” and “myelodysplastic syndromes” with no limitations on language or publication type. We also conducted manual searches of the bibliographies of all included studies [20,21,22,23,24,25,26,27,28,29,30,31] and reviewed articles to identify any remaining studies. We utilized Zotero version 6.0.4 (Corporation for Digital Scholarship, Vienna, VA, USA) to extract duplicate research.

### 2.2. Study Selection

Two reviewers (M.K., D.S.) initially selected studies based on titles and abstracts. Next, we obtained the full texts of studies that met the incorporation criteria for evaluation. Any inconsistencies referring to the studies were resolved by consensus in consultation with two other reviewers (A.P., A.S.).

### 2.3. Inclusion Criteria

Studies were qualified if they met the following criteria: (1) they were observational studies, including case–control, cohort, or cross-sectional studies [36] and (2) they evaluated hepcidin levels in any biological fluids with various laboratorial techniques exclusively in patients with MDS. Neither the stage of the disease, its severity, nor any treatment modalities were used as exclusion criteria for this systematic review.

### 2.4. Exclusion Criteria

Studies were rejected if they involved patients with diseases other than MDS. Observational studies remain prevalent in the literature when assessing serum hepcidin concentrations in patient groups with MDS that coexist with other oncohematological diseases, predominantly acute leukemia. In these studies, the number of patients with MDS did not constitute the majority [37,38,39,40]. The inclusion of such manuscripts may pose a challenge when drawing valid conclusions; the papers referenced were the focus of our prior systematic review [32].

Insufficient data on hepcidin, which included articles focusing on levels of the inactive precursor prohepcidin rather than its active form (hepcidin) and studies published in non-English languages, constituted additional exclusion criteria. Clinical trials, case reports, reviews, editorials, comments, position articles, guidelines, chapters of books, conference proceedings, and non-human studies were also eliminated.

### 2.5. Data Extraction

Significant data from the included studies were extracted by four reviewers (M.K., D.S., A.P., A.S.). Data were gathered from each suitable study based on the following criteria: general study information (first author, study design, year of publication, and study location), participant characteristics (sample size, age, sex, diagnosis, and therapeutic modalities), details relating to the assessment of hepcidin (type of biomaterial, measurement time with corresponding detection method, and hepcidin levels), and, lastly, each study’s main findings. All data were taken directly from the published research; we did not contact the corresponding authors to collect further information.

### 2.6. Quality Assessment

We evaluated the methodological quality of the included research using the Newcastle–Ottawa (NOS) scale for case–control and cohort studies [41]. NOS scores are recommended for the assessment of non-randomized studies [42]. The original NOS consists of eight items distributed across three areas: selection (total score 4), comparability (total score 2), and exposure for case–control studies or outcome for cohort studies (total score 3). The highest total score is nine points. The NOS, as adapted for cross-sectional studies, consists of eight items in three areas: selection (total score 5), comparability (total score 2), and outcome (total score 3), with a maximum total of 10 points [43]. A total score of 3 or less is considered low quality, 4 to 6 is considered moderate quality, and 7 to 9 (10 in cross-sectional studies) indicates high quality [44].

This review’s assessment process consisted of two steps. Initially, two reviewers (M.K., D.S.) conducted quality control and subsequently two separate reviewers (A.P., A.S.) proofread the text to identify any inconsistencies. Thereafter, any discrepancies were discussed and resolved among the four reviewers (M.K., D.S., A.P., A.S.).

## 3. Results

### 3.1. Literature Search Results

**Figure 1:**
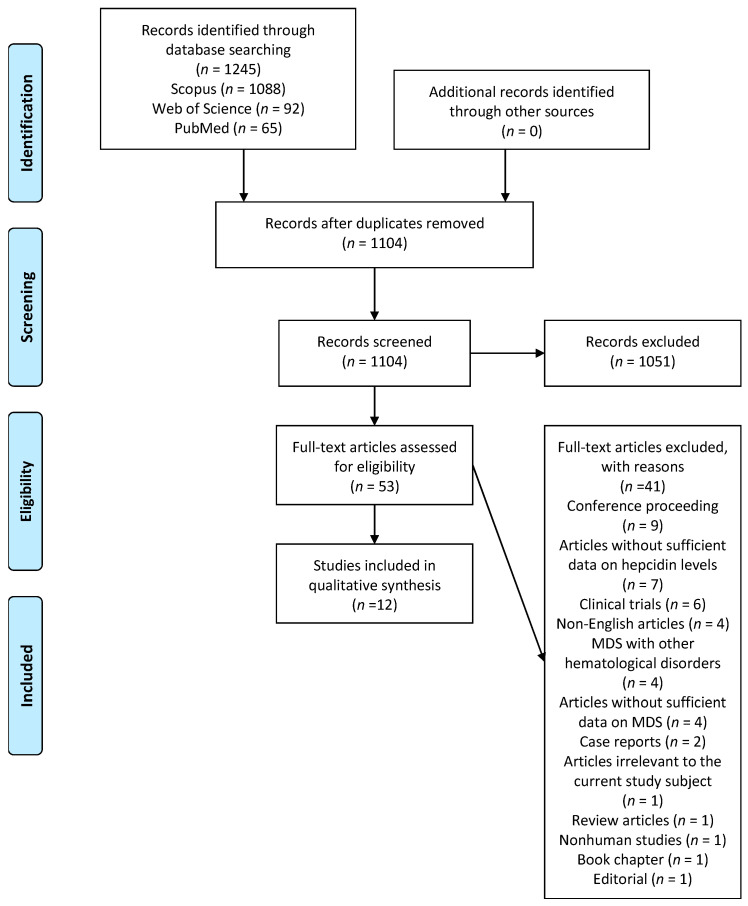
PRISMA flowchart of the search process.

Figure 1, based on PRISMA, showcases the results obtained from the bibliographic database search. We identified 1245 citations, with the majority, 1088 (87%), being found in the Scopus database. After removing duplicates, 1104 citations remained. A further analysis of titles and abstracts deemed 1051 citations unsuitable; thus, they were rejected. The remaining 53 citations were subjected to a thorough full-text investigation. Ultimately, 12 references were included in this systematic review [20,21,22,23,24,25,26,27,28,29,30,31]. The primary reason for excluding full-text citations was a lack of complete data on hepcidin levels in patients with MDS. Detailed bibliographic data of the 41 excluded citations are supplied in Table S3 within the Supplementary Materials. Out of the twelve qualified observational studies, we identified four case–control studies [20,21,22,23], six cohort studies [24,25,26,27,28,29], and two cross-sectional studies [30,31].

### 3.2. Quality of the Included Studies

We used NOS guidelines to assess the quality of the included studies. Overall, one study was classified as high-quality (NOS = 7) and eleven studies as moderate-quality (NOS = 4–6). The median NOS score for all 12 studies was 6. A detailed NOS evaluation along with the number of points (stars) for each study can be found in the Supplementary Materials (Tables S4–S6).

### 3.3. Description of Case–Control Studies

We included four case–control studies in our systematic review [20,21,22,23]. These studies were conducted in Italy [20], China [21,22] and Egypt [23] between 2011 [20] and 2014 [23]. Two studies included a study period covering the years 2009–2012 [21] and 2011-2013 [22]. Three studies were conducted in one center [21,22,23] and one study was conducted in three centers [20]. The number of MDS patients ranged from 21 [23] to 113 [20]. The total number of MDS patients in all four case–control studies was 314 [20,21,22,23]. Men constituted the majority of MDS patients in three studies [21,22,23], except in the study by Santini et al. [20], where more than twice as many women than men were present. The average age of MDS patients ranged from 50 years [22] to 72.8 years [20]. In one study, the authors reported a mean age of 56 years without specifying whether this included MDS patients or controls [23]. Although all included case–control studies stated that MDS patients were adults [20,21,22,23], the study by Cui et al. [22] also included adolescents, with a very broad age range of MDS patients (16–96 years). This finding holds significant importance in understanding serum hepcidin levels.

When focusing on the subtypes of the disease, a considerable heterogeneity among MDS patients was apparent. This can most likely be attributed to the use of different MDS classification systems, which have changed overtime. For example, Santini et al. [20] used the MDS classification according to the World Health Organization (WHO) from 2002 [45], and Gu et al. [21] and Rui et al. [22] used the WHO classification from 2008 [46]. Additionally, Cui et al. [22] provided the WHO classification for only 70 patients out of 107 included in the study (65%). The study conducted by El Husseiny et al. [23] involved patients with hypoplastic MDS (hMDS), which is not currently recognized as a separate subtype of MDS due to significant differences in the biology of hMDS and other types of the disease [47,48].

Three case–control studies included MDS patients who received transfusions [20,21,23]. In Santini et al.’s study [20], less than 40% of patients received transfusions, and in the Gu et al.’s study [21], almost 70% of patients received transfusions. Only in one study [22] did MDS patients not receive transfusions. Data regarding the treatment of MDS patients were actually quite sparse. In two studies, chelation treatment was not received by MDS patients [20,23], while in the other two studies, the method of treatment was not specified [21,22]. None of the patients were treated with hematopoietic cell transplantation (HCT) [20,21,22,23].

In three included studies [20,21,22], the authors used the International Prognostic Scoring System (IPSS) to assess the prognosis of MDS patients [49], with the largest population being low-risk MDS patients.

The number of participants included in the control groups ranged from 13 [23] to 54 [20]; a male predominance was observed. The total number of control participants in all four studies was 135 [20,21,22,23]. However, it is worth noting that the control group was not correctly matched to the study group, especially in terms of age. In the included studies [20,21,22], the control group was much younger than the MDS patients; this may influence the results of hepcidin levels, especially in women [50]. The data described in this section are presented in Table 1.

### 3.4. Hepcidin Levels in MDS Patients—Data from Case–Control Studies

All four studies [20,21,22,23] measured serum hepcidin levels, with one study [21] utilizing bone marrow as the biological material. Three studies used enzyme-linked immunosorbent assay (ELISA) [21,22,23] and one study used the surface-enhanced laser desorption/ionization time-of-flight mass spectrometry (SELDI-TOF MS) method [20]. To standardize the hepcidin findings evaluated in the study [20], results initially given in nM/L were converted to ng/mL, using the conversion factor that 1 nM of serum hepcidin is equal to 2.79 ng/mL [50].

Three studies found no significant difference in hepcidin levels between MDS patients and control participants, whether measured in serum [20,23] or bone marrow [21]. In the study by Cui et al. [22], the authors observed higher hepcidin levels in MDS patients than in controls. The main difference among these studies is that Cui et al. [22] was the only study where patients did not receive transfusions.

Noteworthy observations from case–control studies included hepcidin levels varying with MDS type. Both Santini et al. [20] and Gu et al. [21] found the lowest hepcidin levels in refractory anemia with ring sideroblasts (RARS) and the highest in refractory anemia with excess of blasts (RAEB). Contrary to the aforementioned studies, El Husseiny et al. did not establish a firm conclusion [23], possibly pertaining to the use of a different MDS classification than the other authors [20,21] as well as the relatively small cohort of MDS patients. Only one study [21] revealed that high-risk MDS was associated with high hepcidin levels, but it did not specify what biological material was involved (serum or bone marrow) to obtain such an observation.

It is also worth noting that hepcidin levels in MDS patients and controls differed significantly based on the study and laboratory method used. This emphasizes the need for the standardization of analytical methods when determining hepcidin levels in biological material—something that has been postulated for years [51,52]. Hepcidin levels in MDS patient sera ranged from 14.81 ng/mL [20] to 301.61 ng/mL [21], which may be related to the laboratory method used (SELDI-TOF MS vs. ELISA). Nevertheless, a large range of serum hepcidin levels in MDS patients was also found using the ELISA test, as observed in values ranging from 55.8 ng/mL [23] to 301.61 ng/mL [21]. Similarly, a high variability of serum hepcidin levels occurred in controls, ranging from 11.72 ng/mL [20] to 55.9 ng/mL [22]. In Gu et al. [21], particularly high hepcidin levels were seen in the bone marrow of healthy volunteers with a mean value of 335.71 ng/mL.

All case–control studies [20,21,22,23] had very high ferritin levels, verifying laboratory findings of iron overload in MDS patients. The case–control studies included in this systematic review also confirmed that MDS hepcidin levels were related to transfusion, iron stores in the body, and inflammation [20,21]. These data are summarized in Table 2.

### 3.5. Description of Cohort Studies

Six cohort studies published between 2011 [24] and 2021 [29] were part of our systematic review [24,25,26,27,28,29]. Three studies involved MDS patients from at least two European and Asian countries [24,28,29]. The remaining three studies [25,26,27] focused on MDS patients from one center in Germany [25] and China [26,27]. All studies, except one [24], defined a research period, which in all cases extended until 2014 [25,26,27,28,29]. The follow-up lasted between 3 months [24] and 6.6 years [29].

The included number of MDS patients ranged from 19 [24] to 256 [29], totaling 547 participants and exhibiting a male predominance [24,25,26,27,28,29]. The mean age of MDS patients ranged from 63 years [26] to 74 years [29]. One study neither specified the gender nor the age of the MDS patients [27].

Similarly to the case–control studies [20,21,22,23], the included cohort studies were characterized by a large difference in patient MDS classification systems [24,25,26,27,28,29]. Nevertheless, an analysis of the cohort studies shows that refractory cytopenia with multilineage dysplasia (RCMD) was the most common subtype of MDS studied [24,25,28,29]. IPSS was used in four of the six cohort studies [24,26,28,29], with a predominance of low-risk patients. In one study [28], MDS classification and IPSS were determined in 92% of patients.

Data on the therapy used in the cohort studies [24,25,26,27,28,29] seem to be more complete than in the case–control studies [20,21,22,23]. In four studies, MDS patients received chelation therapy (deferasirox or deferoxamine) [24,27,28,29]. All cohort studies [24,25,26,27,28,29] presented data on transfusions, including their number, the number of patients who received transfusions, and the average number of units transfused. However, none of the cohort studies seemed to connect MDS progression to acute myeloid leukemia (AML) and its potential association with hepcidin levels. HCT was not used in any of the cohort studies [24,25,26,27,28,29]. Data are summarized in Table 3.

### 3.6. Hepcidin Levels in MDS Patients—Data from Cohort Studies

In all six cohort studies, hepcidin levels were measured in serum [24,25,26,27,28,29], with most studies using ELISAs [24,25,26,27]. As detailed in Table 4, hepcidin levels varied dramatically between studies. Overall, MDS patients from cohort studies also had elevated ferritin levels, suggesting an iron overload. These studies established a correlation between serum hepcidin levels and ferritin levels [25,26]. However, the clarity of this relationship is not consistent and depends on factors such as the genetic status of MDS patients [26].

The analysis of the included cohort studies yielded the following key findings: (1) serum hepcidin levels were increased by chelation treatment in MDS patients [24,27]; (2) high serum hepcidin levels characterized high-risk MDS [27] and were associated with worse survival in MDS patients. High serum hepcidin levels in MDS patients were related to (1) RAEB subtype [25] and (2) a lack of mutations in the splicing factor 3B subunit 1 (*SF3B1*) gene [26]. Transfusions influenced the behavior of serum hepcidin levels, leading to an increase over time in transfusion-dependent MDS patients [29] and a decrease over time in transfusion-independent MDS patients [28]. 

### 3.7. Description of Cross-Sectional Studies and Data on Hepcidin

Only two cross-sectional studies were included in our systematic review [30,31]. Hence, their significance with respect to hepcidin’s role in MDS patients is described in the present subsection. These studies, conducted in Europe and published between 2013 [30] and 2017 [31], focused on a relatively small group of MDS patients, totaling a total of 101 individuals, predominantly men, with an average age close to 70 years. The RARS subtype of MDS patients predominated in this cohort. One study [31] used the IPSS and found mainly low-risk MDS patients. These studies involved patients receiving transfusion [30,31] and/or chelation therapy [31]. Both studies used mass spectrometry (MS) methods [30,31] to determine hepcidin levels in serum. Ambaglio et al. [30] confirmed that patients with a mutation in the SF3B1 gene have low hepcidin levels. A very similar observation was made by Zhu et al. [26] in the cohort study discussed in the preceding subsections. In the last study, the authors demonstrated that MDS was associated with higher serum hepcidin levels compared to thalassemia and sickle cell anemia (SCA) [31]. These data are summarized in Table 3 and Table 4.

### 3.8. Relationship between Hepcidin Levels in MDS and Transfusions

While the transfusion–hepcidin axis problem in MDS patients has been briefly discussed in the subsection on cohort studies, this section of our manuscript integrates and summarizes these data, irrespective of the type of study.

Primarily, the majority of the included studies showed elevated serum hepcidin levels in transfusion-dependent MDS patients compared to their non-transfusion-dependent counterparts [20,25,29] or control participants [20]. A positive relationship was also observed between hepcidin levels and the number of units transfused [24,28]. Only one study [21] demonstrated a different relationship from the others [20,25,29], indicating lower hepcidin levels in transfusion-dependent MDS patients. However, the authors did not specify whether this observation pertained to hepcidin levels in serum or bone marrow. Nevertheless, our systematic analysis of observational studies clearly indicates a positive relationship between transfusions and serum hepcidin levels in MDS patients.

### 3.9. Relationship between Hepcidin Levels and Ferritin Levels in MDS

In this systematic review, the included studies demonstrated hyperferritinemia among patients diagnosed with MDS, indicating iron overload. Across the selected studies, some demonstrated a relationship between hepcidin and ferritin levels, revealing a positive association in MDS patients [25,28,30]. In Zhu et al. [26], hepcidin and ferritin exhibited a positive correlation, but solely in patients lacking the *SF3B1* mutation.

## 4. Discussion

To our knowledge, this systematic review provides novel insights into the relationship between hepcidin and myelodysplastic syndromes (MDS).

Adhering to the rigorous methodology outlined in the Preferred Reporting Items for Systematic Reviews and Meta-Analyses (PRISMA) principles and with a specific focus on observational studies, we successfully summarized the current knowledge regarding the correlation between hepcidin and MDS. Despite the varying clinical characteristics observed in MDS patients and the differences in laboratory methods detailed in each paper, we drew the following conclusions: (1) although high serum hepcidin levels are linked to MDS, most studies generally found no significant difference in these levels between patients and healthy individuals; (2) serum hepcidin levels are specific to MDS type; (3) serum hepcidin levels in MDS are strongly associated with transfusions and the patient’s genetic status; (4) high-risk MDS is associated with high serum hepcidin levels. Our systematic review further validated the relationship between serum hepcidin levels in MDS and ferritin, commonly considered the primary laboratory marker of iron overload [53].

Naturally, variability was observed among the studies, primarily concerning MDS classification systems. It should, however, be emphasized that the included studies primarily focused on low-risk adult patients diagnosed with refractory cytopenia with multilineage dysplasia (RCMD) who were undergoing blood transfusions and/or iron chelation. This enables us to draw overarching conclusions regarding the role of hepcidin in MDS. We not only suggest a potential usefulness of this protein in elucidating the etiopathogenesis of iron excess associated with MDS, but also highlight its potential value in other stages of MDS patient management, including the prognosis of disease progression.

Iron overload is undoubtedly a towering problem that every clinician must contend with daily when caring for MDS patients [54]. This issue is widespread and primarily arises due to challenges in utilizing iron effectively during ineffective erythropoiesis, compounded by frequent transfusions. It is part of the inherent landscape of oncohematology clinics dealing with patients with MDS [55]. Thus, it is not surprising that scientists have diligently sought solutions to understand iron metabolism in such patients and how to effectively control it. These investigations have prompted researchers to dedicate considerable attention to comprehending the intricacies of iron metabolism in these patients. The latest achievements in the field of iron metabolism come to the rescue, especially the discovery of hepcidin, which sheds a new light on the pathophysiology of iron metabolism imbalance in the course of many diseases, including MDS [56,57]. Hepcidin functions as a precise switch, effectively regulating the absorption and release of iron in the body. It plays a crucial role in protecting the body from iron overload and its consequences [58].

In this manuscript, our primary focus was to conduct a systematic evaluation of hepcidin’s role in MDS. The art of conducting this type of research allowed us to extract detailed data from observational studies. Despite variations among the manuscripts, our efforts led to the comprehensive summarization of extensive and diverse data on hepcidin’s role in MDS. While analyzing the results, certain findings also came as a surprise to us. A significant observation emerged: although the included studies were nearly consistent in finding no statistically significant difference in serum hepcidin levels between MDS patients and healthy volunteers, MDS patients consistently exhibited elevated serum hepcidin levels. Nevertheless, the design of case–control studies deserves critical attention, especially in terms of control group selection. In the analyzed studies, the controls were often much younger than the MDS patients [20,21]; hence, it is difficult to draw conclusions on the relationship between hepcidin and MDS based solely on studies planned this way. This was a critical factor influencing the final conclusions of the case–control studies. Consequently, real dependencies require a more precise selection of population-based controls.

While taking into account the results we presented, a vital conclusion drawn from this systematic review was the need to plan and conduct a cohort study. Our study allows for a more global view of the role of hepcidin in MDS and facilitates an understanding of the elements needed by subsequent researchers to plan research not only on hepcidin, but on other parameters of iron metabolism. First of all, we would like to point out that hepcidin levels are strongly dependent on the type of MDS, which has been confirmed in both case–control and cohort studies [20,21,25]. Ideally, future studies will also take into account the MDS genetic status of patients, with particular attention drawn to the splicing factor 3B subunit 1 (*SF3B1*) gene mutations, which appear to strongly influence the iron status of MDS patients. Said relationship was also confirmed in two types of observational studies—a cohort study and a cross-sectional study [26,30]. Previous findings have revealed an association between the *SF3B1* gene mutation in MDS patients and elevated plasma ERFE levels, potentially causing a decrease in hepcidin synthesis. This, therefore, may result in a clinically significant iron overload in a specific group of MDS patients [59]. Hence, altering the ERFE–hepcidin axis could present itself as a potential therapeutic avenue for managing this complication in MDS. Furthermore, the prospect of utilizing hepcidin or minihepcidin agonists to prevent iron overload in MDS patients is an intriguing concept [60,61]. Said molecules are currently undergoing clinical trials, primarily in patients with β-thalassemia [62,63,64]. While their application in MDS remains plausible, additional research in this area is necessary. Additionally, it also seems necessary to take therapy into account, including data on transfusions. The relationship between high serum hepcidin levels and MDS patient outcomes is also in need of investigation. Some of the analyzed studies suggested a potential relationship between high serum hepcidin levels and disease severity as well as patient survival, but the data supporting this connection are more than sparse [21,25]. For these reasons, we postulate that one of the important results of our systematic review is the need to absolutely take into account the above-described cofactors that affect the levels of hepcidin in MDS. This will certainly allow for a more realistic presentation of the role of hepcidin as a prognostic factor in patients diagnosed with MDS.

Despite the fact that we adhered to the fine-grained methodology of systematic reviews, it is essential to address the limitations inherent in this study. First, our study was based on a relatively small number of MDS patients and control participants. For this reason, we recommend conducting research on a larger scale, considering greater patient and control cohorts. Nevertheless, it is crucial to bear in mind that MDS is a rare oncological disease, necessitating the simultaneous involvement of multiple centers for comprehensive research in this field. Furthermore, we noted a considerable clinical diversity among the patients during our observations. On the other hand, a natural feature of MDS is the different biology and clinical characteristics among affected individuals. Laboratory differences, mainly resulting from the use of different analytical methods to assess hepcidin levels in serum, can now be eliminated due to the availability of well-validated, extremely sensitive, and precise tests on the market, e.g., based on enzyme-linked immunosorbent methods (ELISA). However, keeping the above limitations in mind, we did not conduct a meta-analysis, but focused on systematically indicating the current state of knowledge about the hepcidin–MDS axis, emphasizing future research directions.

## 5. Conclusions

To conclude, our study, based on a systematic review of observational studies, showed a potential association of MDS with hepcidin, but detailed relationships remain to be investigated. Nevertheless, hepcidin emerges as a crucial and promising element in the pathophysiology of MDS with its potential use serving as a likely predictor of unfavorable outcomes. Whether hepcidin will evolve into a diagnostic and therapeutic element in patients with MDS remains a mystery.

## Figures and Tables

**Table 1:** Demographic and clinical characteristics of the case–control studies included in this systematic review.

First Author, Year	Study Period	Region of Origin	Number of Patients	Mean or Median Age of Patients [Years]	MDS Subtypes	IPSS	Number of Controls	Mean or Median Age of Controls [Years]	Control Group Selection
Santini, V., 2011 [20]	ND	Europe (Italy)	113 (W: 36, M: 77)	72.8	RAEB (*n* = 32)	RAEB: Intermediate-2 (*n* = 17) Intermediate-1 (*n* = 11) High risk (*n* = 4)	54 (W: 21, M: 33)	34.8	(1) Sex-matched controls. (2) Normal iron status.
					RA (*n* = 31)	RA: Low risk (*n* = 20) Intermediate-1 (*n* = 10) Intermediate-2 (*n* = 1)			
					RCMD (*n* = 19)	RCMD: Low risk (*n* = 12) Intermediate-1 (*n* = 7)			
					RARS (*n* = 9)	RARS: Low risk (*n* = 9)			
					Unclassified (*n* = 8)	-			
					5q-syndrome (*n* = 7)	5q-syndrome: Low risk (*n* = 6) Intermediate-1 (*n* = 1)			
					CMML (*n* = 7)	CMML: Low risk (*n* = 4) Intermediate-1 (*n* = 2) Intermediate-2 (*n* = 1)			
Gu, S., 2013 [21]	2009–2012	Asia (China)	73 (W: 34, M: 39)	62	RCMD (*n* = 21)	Low risk (*n* = 46) High risk (*n* = 27)	28 (W:10, M:18)	39	(1) Healthy individuals.
					RCUD (*n* = 11)				
					RAEB-1 (*n* = 11)				
					RAEB-2 (*n* = 10)				
					MDS-U (*n* = 8)				
					RARS (*n* = 7)				
					5q-syndrome (*n* = 5)				
Cui, R., 2014 [22]	2011–2013	Asia (China)	107 (W:41, M:66)	50	RCMD/RCMDRS (*n* = 34)	Intermediate-1 (*n* = 60) Intermediate-2 (*n* = 24) No data (*n* = 9) Low risk (*n* = 8) High risk (*n* = 6)	40 (W:22, M:18)	40	(1) Normal iron status.
					RAEB-1/RAEB-2 (*n* = 25)				
					RA/RARS (*n* = 9)				
					Unclassified (*n* = 1)				
					5q-syndrome (*n* = 1)				
El Husseiny, N.M., 2014 [23]	ND	Africa (Egypt)	21 (W:10, M:11)	56 ^1^	RCMD (*n* = 8)	ND	13 (W:6, M:7)	56 ^1^	(1) Age- and sex-matched controls.
					Hypoplastic MDS (*n* = 7)				
					RAEB (*n* = 6)				

^1^ The authors reported the mean age but did not specify whether it applied to patients, controls, or a combination of these two groups. Abbreviations: CMML = chronic myelomonocytic leukemia; IPSS = International Prognostic Scoring System; M = men; MDS = myelodysplastic syndromes; MDS-U = unclassified myelodysplastic syndromes; ND = not determined; RA = refractory anemia; RAEB = refractory anemia with excess blasts; RAEB-1 = refractory anemia with excess blasts type 1; RAEB-2 = refractory anemia with excess blasts type 2; RARS = refractory anemia with ringed sideroblasts; RCMD = refractory cytopenia with multilineage dysplasia; RCMDRS = RCMD with ring sideroblasts; RCUD = refractory cytopenia with unilineage dysplasia; W = women.

**Table 2:** Main results from case–control studies included in this systematic review.

First Author, Year	Hepcidin Assay	Type of Biological Material	Mean or Median Hepcidin Levels in MDS Patients [ng/mL]	Mean or Median Hepcidin Levels in Controls [ng/mL]	Transfusions	Iron Chelation Therapy	Mean or Median Ferritin Levels in MDS Patients [μg/L]	Main Study Results Related to Hepcidin Levels
Santini, V., 2011 [20]	SELDI- TOF MS	Blood (serum)	All patients: 14.81 ^1^	11.72 ^1^	Transfused patients (*n* = 45)	No iron chelation	All patients: 515	(1) Hepcidin levels did not differ significantly between patients and controls. (2) Hepcidin levels varied significantly across MDS subtypes, with the lowest levels seen in RARS and the highest in RAEB. (3) MDS hepcidin levels were related to transfusions, iron overload (IO), and inflammation.
			RAEB: 31.55		RAEB (*n* = 18)		RAEB: 661	
			RA: 9.65		RA (*n* = 9)		RA: 368	
			RCMD: 10.69		RCMD (*n* = 5)		RCMD: 420	
			RARS: 3.99		RARS (*n* = 3)		RARS: 725	
			Unclassified: 16.91		Unclassified (*n* = 4)		Unclassified: 580	
			5q-syndrome: 18.47		5q-syndrome (*n* = 2)		5q-syndrome: 1364	
			CMML: 28.01		CMML (*n* = 4)		CMML: 289	
Gu, S., 2013 [21]	ELISA (Cusabio Biotech, Wuhan, China)	Blood (serum) Bone marrow (BM)	All patients: 301.61 (serum), 265.66 (BM)	335.71 (BM)	Transfused patients (*n* = 51)	ND	All patients: 621	(1) BM hepcidin levels did not differ significantly between patients and controls. (2) Hepcidin levels did not differ significantly between serum and BM in MDS patients. (3) Hepcidin levels varied significantly across MDS subtypes with the lowest levels seen in RARS and the highest in RAEB-1. ^2^ (4) High risk MDS patients have higher hepcidin levels than low risk patients. ^2^ (5) MDS hepcidin levels are related to transfusions, IO, and inflammation. ^2^
			RCMD: 237.16 ^2^				RCMD: 1444	
			RCUD: 265.66 ^2^				RCUD: 66	
			RAEB-1: 335.71 ^2^				RAEB-1: 985	
			RAEB-2: 301.09 ^2^				RAEB-2: 629	
			MDS-U: 306.08 ^2^				MDS-U: 78	
			RARS: 105.40 ^2^				RARS: 921	
			5q-syndrome: 299.49 ^2^				5q-syndrome: 598	
Cui, R., 2014 [22]	ELISA (DRG Instruments, Marburg, Germany)	Blood (serum)	81.7	55.9	No transfusions	ND	450 ^3^	(1) Hepcidin levels were higher in patients than in controls.
El Husseiny, N.M., 2014 [23]	ELISA (DRG Instruments GmbH, Marburg, Germany)	Blood (serum)	All patients: 55.8	19.9	3-9 blood units (median: 6 blood units)	No iron chelation	All patients: 539.14 ^3^	(1) Hepcidin levels did not differ significantly between patients and controls. (2) Hepcidin levels did not vary across MDS subtypes. (3) MDS hepcidin levels were not related to transfusions and IO.
			RCMD: 66.6				RCMD: 527.3	
			Hypoplastic MDS: 22.6				Hypoplatsic MDS: 434.5	
			RAEB: 80.1				RAEB: 676.8	

^1^ The authors originally measured hepcidin levels using the unit nM/L; however, for standardization, the results in Table 2 are expressed in ng/mL. The following conversion factor was used according to Galesloot TE et al. [50]: 1 nM serum hepcidin equals 2.79 ng/mL. ^2^ The authors did not specify which form of hepcidin (serum, bone marrow) their results referred to. ^3^ The authors originally measured ferritin levels using the unit ng/mL; however, for standardization, the results in Table 2 are expressed in μg/L. Abbreviations: BM = bone marrow; CMML = chronic myelomonocytic leukemia; ELISA = enzyme-linked immunosorbent assay; MDS = myelodysplastic syndromes; MDS-U = unclassified myelodysplastic syndromes; ND = not determined; RA = refractory anemia; RAEB = refractory anemia with excess blasts; RAEB-1 = refractory anemia with excess blasts type 1; RAEB-2 = refractory anemia with excess blasts type 2; RARS = refractory anemia with ringed sideroblasts; RCMD = refractory cytopenia with multilineage dysplasia; RCUD = refractory cytopenia with unilineage dysplasia; SELDI-TOF MS = surface-enhanced laser-desorption/ionization time-of-flight mass spectrometry.

**Table 3:** Demographic and clinical characteristics of the cohort and cross-sectional studies included in this systematic review.

First Author, Year	Study Period	Region of Origin	Number of Patients	Mean or Median Age of Patients [Years]	MDS Subtypes	IPSS	Follow-Up
Ghoti, H., 2011 [24]	ND	Asia (Israel) North America (USA)	19 (W: 12, M: 7)	68	RCMD (*n* = 11)	Intermediate (*n* = 11) Low risk (*n* = 8)	3 months
					RA (*n* = 5)		
					RARS (*n* = 3)		
Zipperer, E., 2013 [25]	2009–2010	Europe (Germany)	89 (W: 37, M: 52)	71	RCMD (*n* = 38)	ND	18 months
					RAEB-2 (*n* = 14)		
					RAEB-1 (*n* = 8)		
					RCMDRS (*n* = 7)		
					5q-syndrome (*n* = 7)		
					CMML-2 (*n* = 6)		
					CMML-1 (*n* = 4)		
					RARS (*n* = 3)		
					RA (*n* = 2)		
Zhu, Y., 2016 [26]	2008–2014	Asia (China)	52 (W: 21, M: 31)	All patients: 63 *SF3B1* mutation: 64 No mutation: 61	RARS (*n* = 23)	Intermediate-1 (*n* = 28) Low risk (*n* = 14) Intermediate-2 (*n* = 8) High risk (*n* = 2)	18 months
					RCMDRS (*n* = 20)		
					RAEB-1 (*n* = 6)		
					RAEB-2 (*n* = 3)		
Gu, S., 2017 [27]	2010–2014	Asia (China)	22 (W: ND, M: ND)	ND	RCUD/RARS/5q-syndrome/RCMD (*n* = 15) (low-risk MDS)	ND	26 weeks
					RAEB-1/RAEB-2 (*n* = 7) (high-risk MDS)		
de Swart, L., 2018 [28]	2008–2010	Europe (The Netherlands, United Kingdom, Sweden, Romania, Greece, Czechia)	109 (W: 45, M: 64)	73	RCMD ^1^ (*n* = 37)	Low risk (*n* = 47) ^1^ Intermediate-1 (*n* = 41) Unknown (*n* = 12)	5.8 years
					RARS (*n* = 30)		
					RA (*n* = 18)		
					RAEB (*n* = 7)		
					RCMDRS (*n* = 4)		
					5q-syndrome (*n* = 4)		
Hoeks, M., 2021 [29]	2008	Europe, Asia (The Netherlands, United Kingdom, Greece, Sweden, Romania, Czech Republic, Austria, Croatia, Denmark, France, Germany, Israel, Italy, Poland, Portugal, Serbia, Spain)	256 (W: 87, M: 169)	74	RCMD (*n* = 114)	Low risk (*n* = 144) Intermediate-1 (*n* = 75) Unknown (*n* = 36) Intermediate-2 (*n* = 1)	6.6 years
					RARS (*n* = 56)		
					RA (*n* = 45)		
					RAEB-1 (*n* = 16)		
					RCMDRS (*n* = 10)		
					5q-syndrom (*n* = 10)		
					Unclassified (*n* = 5)		
Ambaglio, I., 2013 [30]	ND	Europe (Italy)	76 (W: 36, M: 40)	67	RARS/RCMDRS (*n* = 26)	ND	-
					RAEB-1/RAEB-2 (*n* = 23)		
					RA/RCMD (*n* = 22)		
					RARS-T (*n* = 5)		
Montalembert, M., 2017 [31]	2012–2014	Europe (France)	25 (W: 8, M: 17)	69.5	RARS (*n* = 12)	Low risk (*n* = 15) Intermediate-1 (*n* = 10)	-
					RA (*n* = 6)		
					MDS/MPN (*n* = 2)		
					RAEB-1 (*n* = 2)		
					Unclassified (*n* = 2)		
					5q-syndrome (*n* = 1)		

^1^ Data were available for 100 patients. Abbreviations: CMML = chronic myelomonocytic leukemia; CMML-1 chronic myelomonocytic leukemia type 1; CMML-2 chronic myelomonocytic leukemia type 2; IPSS = International Prognostic Scoring System; M = men; MDS = myelodysplastic syndromes; MDS-RS = myelodysplastic syndromes with ring sideroblast; MDS non-RS = myelodysplastic syndromes without ring sideroblast; MPN = myeloproliferative neoplasm; ND = not determined; RA = refractory anemia; RAEB = refractory anemia with excess blasts; RAEB-1 = refractory anemia with excess blasts type 1; RAEB-2 = refractory anemia with excess blasts type 2; RARS = refractory anemia with ringed sideroblasts; RARS-T = refractory anemia with ringed sideroblasts associated with marked thrombocytosis; RCMD = refractory cytopenia with multilineage dysplasia; RCMDRS = RCMD with ring sideroblasts; RCUD = refractory cytopenia with unilineage dysplasia; W = women.

**Table 4:** Main results from cohort and cross-sectional studies included in this systematic review.

First Author, Year	Hepcidin Assay	Type of Biological Material	Mean or Median Hepcidin Levels in Patients [ng/mL]	Transfusions					Iron Chelation Therapy	Mean or Median Ferritin Levels in MDS Patients [μg/L]					Main Study Results Related to Hepcidin Levels
Ghoti, H., 2011 [24]	ELISA (in house)	Blood (serum)	545 (before treatment)	Mean number of transfusions: 45.6					Deferasirox (20 mg/kg/day)	1558 ^1^					(1) Hepcidin levels were increased by treatment with deferasirox.
			811 (after treatment)												
Zipperer, E., 2013 [25]	ELISA (DRG Instruments GmbH, Marburg, Germany)	Blood (serum)	All patients: 17.5	Transfused patients (*n* = 41) 2-83 units ^2^					ND	All patients: 876 ^1^					(1) Hepcidin levels varied significantly across MDS subtypes, with the lowest levels seen in RA/RARS and the highest in RAEB 1/2. (2) High hepcidin levels were associated with worse survival.
			RCMD: 17.8							RCMD: 950					
			RAEB-1/RAEB-2: 29.1							RAEB-1/RAEB-2: 1130					
			RCMDRS: 8.7							RCMDRS: 643					
			5q-syndrome: 26.3							5q-syndrome: 1680					
			CMML-1/CMML-2: 16.9							CMML-1/CMML-2: 395					
			RA/RARS: 5.9							RA/RARS: 584					
Zhu, Y., 2016 [26]	ELISA (Bachem, San Carlos, CA, USA)	Blood (serum)	*SF3B1* mutation: 17.3 No mutations: 77.8	Transfused patients: 0 units (*n* = 17) <10 units (*n* = 14) 10–20 units (*n* = 8) >20 units (*n* = 9) No data (*n* = 4)					ND	*SF3B1* mutation: 1088.9 ^1^ No mutation: 575.7					(1) Patients with the *SF3B1* mutation had significantly lower hepcidin levels than patients without the mutation.
Gu, S., 2017 [27]	ELISA (Cusabio Biotech, Wuhan, China)	Blood (serum)	Low-risk MDS ^3^: 284.24 ^4^	2.37 units per month ^6^					Deferoxamine ^7^	Low-risk MDS ^3^: 2182 mg/L ^8^					(1) Hepcidin levels were higher in high-risk MDS patients compared to low-risk MDS. (2) Hepcidin levels were increased by treatment with deferoxamine.
			High-risk MDS ^5^: 336.47												
			Week 0: 301.26												
										High-risk MDS ^5^: 1733 mg/L					
			Week 4: 325.48												
										Week 0: 1946 mg/L					
			Week 26: 340.33							Week 4: 1829 mg/L					
										Week 26: 1721 mg/L					
de Swart, L., 2018 [28]	WCX and TOF-MS	Blood (serum)	At study inclusion: 12.56 ^9^	1 year follow-up: 15.62 ^9^	2 years follow-up: 14.51 ^9^			Transfused patients (*n* = 14)	Patients on deferasirox (*n* = 4) Patients on deferoxamine (*n* = 2)	At study inclusion: 287					(1) Hepcidin levels decreased in transfusion-independent MDS-RS over time.
			TI MDS-RS: 10.60	TI MDS-RS: 9.49	TI MDS-RS: 8.09					MDS-RS: 376					
			TI MDS non-RS: 12.56	TI MDS non-RS: 12.00	TI MDS non-RS: 12.83					MDS non-RS: 246					
			TD MDS-RS: 28.74	TD MDS-RS: 25.67	TD MDS-RS: 14.51					TD: 634					
			TD MDS non-RS: 13.67	TD MDS non-RS: 48.27	TD MDS non-RS: 25.67					TI: 264					
Hoeks, M., 2021 [29]	WCX and TOF-MS	Blood (serum)	At study inclusion: 23.99 ^9^	Visit 1 ^10^: 26.23 ^9^	Visit 2 ^10^: 28.46 ^9^	Visit 3 ^10^: 28.46 ^9^	Visit 4 ^10^: 24.83 ^9^	At study inclusion: Transfused patients (*n* = 62)	Patients on deferiprone/ deferasirox (*n* = 11) Patients on deferoxamine (*n* = 5)	At study inclusion: 488	Visit 1 ^10^: 605	Visit 2 ^10^: 702	Visit 3 ^10^: 822	Visit 4 ^10^: 858	(1) Hepcidin levels were higher in transfusion-dependent non-RS patients compared to non-RS transfusion-independent patients. (2) Hepcidin levels increased over time in transfusion-dependent patients.
			TI MDS-RS: 13.95	TI MDS-RS: 10.88	TI MDS-RS: 10.32	TI MDS-RS: 10.60	TI MDS-RS: 10.88	Visit 1 ^10^: Transfused patients (*n* = 76)		TI MDS-RS: 505	TI MDS-RS: 356	TI MDS-RS: 343	TI MDS-RS: 493	TI MDS-RS: 441	
			TI MDS non-RS: 17.30	TI MDS non-RS: 17.86	TI MDS non-RS: 15.62	TI MDS non-RS: 18.14	TI MDS non-RS: 14.51	Visit 2: Transfused patients (*n* = 73)		TI MDS non-RS: 280	TI MDS non-RS: 274	TI MDS non-RS: 251	TI MDS non-RS: 262	TI MDS non-RS: 263	
			TD MDS-RS: 21.76	TD MDS-RS: 23.44	TD MDS-RS: 25.95	TD MDS-RS: 41.85	TD MDS-RS: 32.64	Visit 3: Transfused patients (*n* = 70)		TD MDS-RS: 919	TD MDS-RS: 1096	TD MDS-RS: 1627	TD MDS-RS: 2104	TD MDS-RS: 2092	
			TD MDS non-RS: 56.92	TD MDS non-RS: 52.45	TD MDS non-RS: 58.59	TD MDS non-RS: 43.80	TD MDS non-RS: 44.64	Visit 4: Transfused patients (*n* = 48)		TD MDS non-RS: 935	TD MDS non-RS: 1145	TD MDS non-RS: 1297	TD MDS non-RS: 1083	TD MDS non-RS:1399	
Ambaglio, I., 2013 [30]	WCX-TOF-MS	Blood (serum)	19.81 ^9^	Transfused patients (*n* = 25)					ND	409 ^1^					(1) Low hepcidin levels in MDS patients with the *SF3B1* mutation.
de Montalembert, M., 2017 [31]	LC-MS	Blood (serum)	36.35	77 units since diagnosis 27 units per year					Patients on deferasirox (*n* = 16)	1611 ^1^					(1) Hepcidin levels were higher in MDS patients compared to thalassemia (1.35 ng/mL) and sickle cell anemia (2.10 ng/mL) patients.

^1^ The authors originally measured ferritin levels using the ng/mL unit; however, for standardization, the results in Table 4 are expressed in μg/L. ^2^ Data were available for 20 patients. ^3^ The authors defined refractory cytopenia with unilineage dysplasia/refractory anemia with ring sideroblasts/5q-syndrome/refractory cytopenia with multilineage dysplasia as a low-risk group. ^4^ The authors originally measured hepcidin levels using the mg/L unit; however, for standardization, the results in Table 4 are expressed in ng/mL. ^5^ The authors defined refractory anemia with excess blasts-1/refractory anemia with excess blasts-2 as a high-risk group. ^6^ Before deferoxamine treatment. ^7^ Patients began deferoxamine treatment when their adjusted serum ferritin (ASF) was over 1000 mg/L and received iron chelation therapy regularly for the next 26 weeks (study follow-up). ^8^ The authors determined adjusted serum ferritin (ASF = serum ferritin [mg/L]/log10 of C-reactive protein [mg/L]). ^9^ The authors originally measured hepcidin levels using the nM/L unit; however, for standardization, the results in Table 4 are expressed in ng/mL. The following conversion factor was used according to Galesloot TE et al. [50]: 1 nM serum hepcidin equals 2.79 ng/mL. ^10^ Visit 1 was 6 months after inclusion in the study; visit 2 was 12 months after inclusion in the study; visit 3 was 18 months after inclusion in the study; visit 4 was 24 months after inclusion in the study. Abbreviations: CMML = chronic myelomonocytic leukemia; CMML-1 chronic myelomonocytic leukemia type 1; CMML-2 chronic myelomonocytic leukemia type 2; MDS = myelodysplastic syndromes; MDS-RS = myelodysplastic syndromes with ring sideroblast; MDS non-RS = myelodysplastic syndromes without ring sideroblast; MPN = myeloproliferative neoplasm; ND = not determined; RA = refractory anemia; ELISA = enzyme-linked immunosorbent assay; LC-MS = liquid chromatography-mass spectrometry; RAEB = refractory anemia with excess blasts; RAEB-1 = refractory anemia with excess blasts type 1; RAEB-2 = refractory anemia with excess blasts type 2; RARS = refractory anemia with ringed sideroblasts; RARS-T = refractory anemia with ringed sideroblasts associated with marked thrombocytosis; RCMD = refractory cytopenia with multilineage dysplasia; RCMDRS = RCMD with ring sideroblasts; RCUD = refractory cytopenia with unilineage dysplasia; WCX = weak cation exchange chromatography; TD = transfusion-dependent; TI = transfusion-independent; TOF-MS = time-of-flight mass spectrometry; WCX-TOF-MS = weak cation exchange time-of-flight mass spectrometry.

## Supplementary Materials

The following supporting information can be downloaded at: https://www.mdpi.com/article/10.3390/cancers16020332/s1, Table S1: Preferred Reporting Items for Systematic Reviews and Meta-Analyses (PRISMA) checklist; Table S2: PubMed search strategy; Table S3: A complete list of excluded studies along with reasons for exclusion; Table S4: The Newcastle–Ottawa Scale (NOS) for case–control studies; Table S5: The Newcastle–Ottawa Scale (NOS) for cohort studies; Table S6: The Newcastle–Ottawa Scale (NOS) for cross-sectional studies.
