# Supplementary material for: The Role of Hepcidin in Myelodysplastic Syndromes (MDS): A Systematic Review of Observational Studies
Source: Cancers (Basel). 2024 Jan 11;16(2):332. doi: 10.3390/cancers16020332 (PMC10814117; doi:10.3390/cancers16020332)
Supplement: Supplementary file 1 [file cancers-16-00332-s001.zip › cancers-2781494-supplementary.pdf]

Supplementary Materials: The Role of Hepcidin in Myelodysplastic Syndromes (MDS): A Systematic Review of Observational Studies

Artur Słomka [1,*], Anna Pokrzywa [1], Dominika Strzała [1], Maja Kubiaczyk [1], Oliwia Wesolowska [1], Kinga Denkiewicz [1] and Jan Styczyński [2]

[1] Department of Pathophysiology, Nicolaus Copernicus University in Toruń, Ludwik Rydygier Collegium Medicum in Bydgoszcz, 85-094 Bydgoszcz, Poland; 288452@stud.umk.pl (A.P.); 306211@stud.umk.pl (D.S.); 287261@stud.umk.pl (M.K.); 301637@stud.umk.pl (O.W.); 301685@stud.umk.pl (K.D.)
[2] Department of Pediatric Hematology and Oncology, Nicolaus Copernicus University in Toruń, Ludwik Rydygier Collegium Medicum in Bydgoszcz, 85-094 Bydgoszcz, Poland; jstyczynski@cm.umk.pl
* Correspondence: artur.slomka@cm.umk.pl; Tel.: +48-52-585-35-94

Table S1. Preferred Reporting Items for Systematic Reviews and Meta-Analyses (PRISMA) checklist.

Section/Topic	#	Checklist Item	Reported on Page #
		**TITLE**	
**Title**	1	Identify the report as a systematic review, meta-analysis, or both.	**1**
		**ABSTRACT**	
**Structured summary**	2	Provide a structured summary including, as applicable: background; objectives; data sources; study eligibility criteria, participants, and interventions; study appraisal and synthesis methods; results; limitations; conclusions and implications of key findings; systematic review registration number.	**1**
		**INTRODUCTION**	
**Rationale**	3	Describe the rationale for the review in the context of what is already known.	**1–2**
**Objectives**	4	Provide an explicit statement of questions being addressed with reference to participants, interventions, comparisons, outcomes, and study design (PICOS).	**2**
		**METHODS**	
**Protocol and registration**	5	Indicate if a review protocol exists, if and where it can be accessed (e.g., Web address), and, if available, provide registration information including registration number.	**2**
**Eligibility criteria**	6	Specify study characteristics (e.g., PICOS, length of follow-up) and report characteristics (e.g., years considered, language, publication status) used as criteria for eligibility, giving rationale.	**3**
**Information sources**	7	Describe all information sources (e.g., databases with dates of coverage, contact with study authors to identify additional studies) in the search and date last searched.	**2**
**Search**	8	Present full electronic search strategy for at least one database, including any limits used, such that it could be repeated.	**Table S2**
**Study selection**	9	State the process for selecting studies (i.e., screening, eligibility, included in systematic review, and, if applicable, included in the meta-analysis).	**3**
**Data collection process**	10	Describe method of data extraction from reports (e.g., piloted forms, independently, in duplicate) and any processes for obtaining and confirming data from investigators.	**3**
**Data items**	11	List and define all variables for which data were sought (e.g., PICOS, funding sources) and any assumptions and simplifications made.	**3**
**Risk of bias in individual studies**	12	Describe methods used for assessing risk of bias of individual studies (including specification of whether this was done at the study or outcome level), and how this information is to be used in any data synthesis.	**3**
**Summary measures**	13	State the principal summary measures (e.g., risk ratio, difference in means).	**N/A**
**Synthesis of results**	14	Describe the methods of handling data and combining results of studies, if done, including measures of consistency (e.g., I^2) for each meta-analysis.	**N/A**
**Risk of bias across studies**	15	Specify any assessment of risk of bias that may affect the cumulative evidence (e.g., publication bias, selective reporting within studies).	**N/A**
**Additional analyses**	16	Describe methods of additional analyses (e.g., sensitivity or subgroup analyses, meta-regression), if done, indicating which were pre-specified.	**N/A**
		**RESULTS**	
**Study selection**	17	Give numbers of studies screened, assessed for eligibility, and included in the review, with reasons for exclusions at each stage, ideally with a flow diagram.	**4**
**Study characteristics**	18	For each study, present characteristics for which data were extracted (e.g., study size, PICOS, follow-up period) and provide the citations.	**5–14**

**Risk of bias within studies**	19	Present data on risk of bias of each study and, if available, any outcome level assessment (see item 12).	**5**
**Results of individual studies**	20	For all outcomes considered (benefits or harms), present, for each study: (a) simple summary data for each intervention group (b) effect estimates and confidence intervals, ideally with a forest plot.	**N/A**
**Synthesis of results**	21	Present results of each meta-analysis done, including confidence intervals and measures of consistency.	**N/A**
**Risk of bias across studies**	22	Present results of any assessment of risk of bias across studies (see Item 15).	**N/A**
**Additional analysis**	23	Give results of additional analyses, if done (e.g., sensitivity or subgroup analyses, meta-regression [see Item 16]).	**N/A**
		**DISCUSSION**	
**Summary of evidence**	24	Summarize the main findings including the strength of evidence for each main outcome; consider their relevance to key groups (e.g., healthcare providers, users, and policy makers).	**14, 15**
**Limitations**	25	Discuss limitations at study and outcome level (e.g., risk of bias), and at review-level (e.g., incomplete retrieval of identified research, reporting bias).	**16**
**Conclusions**	26	Provide a general interpretation of the results in the context of other evidence, and implications for future research.	**16**
		**FUNDING**	
**Funding**	27	Describe sources of funding for the systematic review and other support (e.g., supply of data); role of funders for the systematic review.	**16**

Abbreviations: N/A = not applicable.

Table S2. PubMed search strategy. A PubMed search was conducted on the 4th of November, 2022.

	Search	Search Details	Records
**1**	hepcidin AND myelodysplastic syndrome	(“hepcidin s”[All Fields] OR “hepcidine”[All Fields] OR “hepcidins”[MeSH Terms] OR “hepcidins”[All Fields] OR “hepcidin”[All Fields]) AND (“myelodysplastic syndromes”[MeSH Terms] OR (“myelodysplastic”[All Fields] AND “syndromes”[All Fields]) OR “myelodysplastic syndromes”[All Fields] OR (“myelodysplastic”[All Fields] AND “syndrome”[All Fields]) OR “myelodysplastic syndrome”[All Fields])	65

Table S3. A complete list of excluded studies along with reasons for exclusion.

Reasons	Number of Papers	References
**Conference proceeding**	9	Ambaglio M, Malcovati L, Della Porta M, et al. Effect of sf3b1 mutation on erythroid marrow activity, transfusion iron overload, and hepcidin levels in patients with myelodysplastic syndrome. Haematologica. 2012;97(1):222.<br>Malcovati L, Della Porta MG, Laarakkers CM, et al. Erythroid Activity, Transfusion Iron Overload, and Hepcidin Levels in Patients with Myelodysplastic Syndrome. Blood. 2008;112(11):2676.<br>Post J, Janetzko A, Kuendgen A, et al. Serum hepcidin levels in patients with myelodysplastic syndrome. Onkologie. 2011;34(6):8–8.<br>De Swart L, Reiniers C, Bagguley T, et al.Transfusions and presence of ringsideroblasts influence hepcidin and ntbi levels in patients with lower-risk myelodysplastic syndromes (mds)—a Report From The European Leukemianet Mds Registry. Leukemia Research. 2015; 39, 106–107.<br>Oarbeascoa G, Redondo S, Morán-Jiménez MJ, et al. Hepcidin and Erythroferrone in the Anemia of Low-Risk Myelodysplastic Syndromes. Blood. 2018; 132 (Supplement 1): 3085.<br>De Swart L, Reiniers C, Bagguley T, et al. Hepcidin and GDF15 Levels during the First 2 Years Follow-up in Patients with Low and Int-1 Risk Myelodysplastic Syndromes (MDS) from the European Leukemianet MDS Registry. Blood. 2014;124(21), 3267.

		Murphy T, Mitra S, Gleeson C, et al. Urinary hepcidin levels are suppressed in low grade myelodysplastic syndrome and may be associated with markers of functional iron deficiency. Haematologica-The Hematology Journal. 2008;93(1):290–291.<br>Winder A, Lefkowitz R, Ghoti H, et al. Urinary Hepcidin Excretion in Patients with Myelodysplastic Syndrome (MDS) and Myelofibrosis (MF). Blood. 2006; 108 (11): 2620.<br>Redondo S, Oarbeascoa G, Moran Jimenez MJ, et al. Hepcidin and erythroferrone (ERFE) are not correlated in the anemia of low-risk myelodysplastic syndromes (MDS). Haematologica. 2018;103(2):183–184.
**Articles without sufficient data on hepcidin levels**	7	Cremers EMP, de Witte T, de Wreede L, et al. A prospective non-interventional study on the impact of transfusion burden and related iron toxicity on outcome in myelodysplastic syndromes undergoing allogeneic hematopoietic cell transplantation. Leuk Lymphoma. 2019;60(10):2404–2414.;<br>Tsang E, Leitch HA. Can iron overload in patients with lower-risk myelodysplastic syndromes be reduced using erythropoiesis-stimulating agents?. Ann Hematol. 2016;95(1):73–78.<br>Israel A, Bornstein G, Gilad L, et al. Clinical and prognostic significance of elevated ferritin levels in hospitalised adults. Postgrad Med J. 2022;98(1162):622–625.<br>Tachibana T, Tanaka M, Numata A, et al. Clinical significance of pre- and 1-year post-transplant serum ferritin among adult transplant recipients. Leuk Lymphoma. 2014;55(6):1350–1356.<br>El Husseiny NM, Matter MM, Sabry RM, et al. Serum prohepcidin level in myelodysplasia. Scand J Clin Lab Invest. 2010;70(5):343–346.<br>Murphy PT, Mitra S, Gleeson M, et al. Urinary hepcidin excretion in patients with low grade myelodysplastic syndrome. British Journal of Haematology. 2009; 144: 451–452.<br>Winder A, Lefkowitz R, Ghoti H, et al. Urinary hepcidin excretion in patients with myelodysplastic syndrome and myelofibrosis. Br J Haematol. 2008;142(4):669–671.
**Clinical trials**	6	Gu S, Chang C, Zhao Y, et al. (2015) Decitabine Treatment Could Ameliorate Primary Iron-Overload in Myelodysplastic Syndrome Patients, Cancer Investigation. 2015;33(4), 98–106.<br>Pirotte M, Fillet M, Seidel L, et al. Erythroferrone and hepcidin as mediators between erythropoiesis and iron metabolism during allogeneic hematopoietic stem cell transplant. Am J Hematol. 2021;96(10):1275–1286.<br>Zheng, Q, Xu H, Song L. et al. Integrated traditional Chinese and conventional medicine in the treatment of anemia due to lower-risk myelodysplastic syndrome: study protocol for a randomized placebo-controlled trial. Trials. 2021;22(1):712.<br>Park S, Kosmider O, Maloisel F, et al. Dyserythropoiesis evaluated by the RED score and hepcidin:ferritin ratio predicts response to erythropoietin in lower-risk myelodysplastic syndromes. Haematologica. 2019;104(3):497–504.<br>Wermke M, Eckoldt J, Götze KS, et al. Enhanced labile plasma iron and outcome in acute myeloid leukaemia and myelodysplastic syndrome after allogeneic haemopoietic cell transplantation (ALLIVE): a prospective, multicentre, observational trial. Lancet Haematol. 2018;5(5):201–210.<br>Garcia-Manero G, Gartenberg G, Steensma DP, et al. A phase 2, randomized, double-blind, multicenter study comparing siltuximab plus best supportive care (BSC) with placebo plus BSC in anemic patients with International Prognostic Scoring System low- or intermediate-1-risk myelodysplastic syndrome. Am J Hematol. 2014;89(9):156–162.
**Non-English articles**	4	Gu S, Song X, Zhao Y, et al. Study of hepcidin level in bone marrow in patients with myelodysplastic syndromes. Journal of Shanghai Jisotong University (Medical Science). 2013;33(1):56–06.<br>Noskova KK, Lishchinskaia AA, Parfenov AI, et al. Risk of development of clinical and pathogenetic features of anemia on the background of basic therapy of inflammatory bowel disease. Eksp Klin Gastroenterol. 2011;(10):12–17.<br>Qin Y, Liu H, Ruan S, et al. Detection of Hepcidin in transfusion dependent myelodysplastic syndrome patients and its clinical significance, Zhonghua Xue Ye Xue Za Zhi. 2011;32(11):758–761.<br>Jomen W, Kuroda H, Arihara Y, et.al. A case of hemosiderin deposition associated with hepatic graft-versus-host disease after peripheral blood stem cell transplantation for acute lymphocytic leukemia. Kanzo/Acta Hepatologica Japonica, 2013;54(11):741–747.
**MDS with other hematological disorders**	4	Kanda J, Mizumoto C, Kawabata H, et al. Clinical significance of serum hepcidin levels on early infectious complications in allogeneic hematopoietic stem cell transplantation. Biol Blood Marrow Transplant. 2009;15(8):956–962.

		Armand P, Sainvil MM, Kim HT, et al. Does iron overload really matter in stem cell transplantation?. Am J Hematol. 2012;87(6):569–572.<br>Armand P, Kim HT, Rhodes J, et al. Iron overload in patients with acute leukemia or MDS undergoing myeloablative stem cell transplantation. Biol Blood Marrow Transplant. 2011;17(6):852–860.<br>Kanda J, Mizumoto C, Kawabata H, et al. Serum hepcidin level and erythropoietic activity after hematopoietic stem cell transplantation. Haematologica. 2008;93(10):1550–4.
**Articles without sufficient data on MDS**	4	Pardanani A, Finke C, Abdelrahman RA, et al. Associations and prognostic interactions between circulating levels of hepcidin, ferritin and inflammatory cytokines in primary myelofibrosis. Am J Hematol. 2013;88(4):312–316.<br>Chen J, Zhong L. Clinical significance of serum hepcidin-25 levels in predicting invasive fungal disease in patients after transplantation. Eur Rev Med Pharmacol Sci. 2013;17(13):1769–1773.<br>Martin-Cabrera P, Hung M, Ortmann E, et al. Clinical use of low haemoglobin density, transferrin saturation, bone marrow morphology, Perl's stain and other plasma markers in the identification of treatable anaemia presenting for cardiac surgery in a prospective cohort studyJournal of Clinical Pathology. 2015;68:923–930.<br>Cangemi G, Pistorio A, Miano M, et al. Diagnostic potential of hepcidin testing in pediatrics. Eur J Haematol. 2013;90(4):323–330.
**Case reports**	2	Lira Zidanes A, Marchi G, Busti F, et al. A Novel ALAS2 Missense Mutation in Two Brothers With Iron Overload and Associated Alterations in Serum Hepcidin/Erythroferrone Levels. Front Physiol. 2020;11:581386.<br>Golombick T, Diamond TH, Manoharan A, et al. Effect of the Ginger Derivative, 6-Shogaol, on Ferritin Levels in Patients With Low to Intermediate-1–Risk Myelodysplastic Syndrome—A Small, Investigative Study. Clinical Medicine Insights: Blood Disorders. 2017;10.
**Articles irrelevant to the current study subject**	1	Zhou Y, Meng JL, Feng L, et al. Abdominal magnetic resonance imaging examination of Tibetan patients with abnormal iron metabolism and a preliminary study of correlations with blood cell analysis. J Int Med Res. 2020;48(3):300060520905483.
**Review articles**	1	Mahesh S, Ginzburg Y, Verma A, Iron overload in myelodysplastic syndromes, Leukemia & Lymphoma 2008;49(3):427–438.
**Nonhuman studies**	1	Miura S, Kobune M, Horiguchi H, et al. EPO-R+ myelodysplastic cells with ring sideroblasts produce high erythroferrone levels to reduce hepcidin expression in hepatic cells. Blood Cells Mol Dis. 2019;78:1–8.
**Book chapter**	1	Enrico A, Flores MG, Kornblihtt L, et al. Anemia in myelodysplastic syndromes, Anemia: Prevalence, Risk Factors and Management Strategies, 2014:65–97.
**Editorial**	1	Kroot JJC, Tjalsma H, Fleming RE, Diagnostic implications of hepcidin in diseases that affect the iron metabolism, Biochimica Clinica, 2013;37(2):108–127.

Table S4. The Newcastle-Ottawa Scale (NOS) for case-control studies.

First Author	Newcastle-Ottawa Scale (NOS)								Total Score
	Selection				Comparability	Exposure			
	Is the case definition adequate?	Representativeness of the cases	Selection of controls	Definition of controls	Comparability of cases and controls on the basis of the design or analysis	Ascertainment of exposure	Same method of ascertainment for cases and controls	Non-response rate	
Santini V, 2011 [20]	★	★	-	★	★	★	★	N/A	**6**
Gu S, 2013 [21]	★	★	-	★	-	★	-	N/A	**4**
Cui R, 2014 [22]	★	★	-	★	★	★	★	N/A	**6**
El Husseiny NM, 2014 [23]	★	★	-	★	★★	★	★	N/A	**7**

Abbreviations: N/A = not applicable.

Table S5. The Newcastle-Ottawa Scale (NOS) for cohort studies.

First Author	Newcastle-Ottawa Scale (NOS)								Total Score
	Selection				Comparability	Exposure			
	Representativeness of the exposed cohort	Selection of the non exposed cohort	Ascertainment of exposure	Demonstration that outcome of interest was not present at start of study	Comparability of cohorts on the basis of the design or analysis	Assessment of outcome	Was follow-up long enough for outcomes to occur	Adequacy of follow up of cohorts	
Ghoti H, 2011 [24]	★	N/A	★	★	N/A	★	★	★	**6**
Zipperer E, 2013 [25]	★	N/A	★	★	N/A	★	★	★	**6**
Zhu Y, 2016 [26]	★	N/A	★	★	N/A	★	★	★	**6**
Gu S, 2017 [27]	★	N/A	★	★	N/A	★	★	★	**6**
de Swart L, 2018 [28]	★	N/A	★	★	N/A	★	★	★	**6**
Hoeks M, 2021 [29]	★	N/A	★	★	N/A	★	★	★	**6**

Abbreviations: N/A = not applicable.

Table S6. The Newcastle-Ottawa Scale (NOS) for cross-sectional studies.

Newcastle-Ottawa Scale (NOS)								
	**Selection**				**Comparability**	**Exposure**		
**First Author**	Representativeness of the sample	Sample size	Non-respondents	Ascertainment of the exposure (risk factor)	The subjects in different outcome groups are comparable, based on the study design or analysis Confounding factors are controlled	Assessment of outcome	Statistical test	**Total Score**
Ambaglio I, 2013 [30]	★	-	N/A	★★	-	★	★	**5**
Montalembert M, 2017 [31]	★	-	N/A	★★	★	★	★	**6**

Abbreviations: N/A = not applicable.
